# Complex Embedding with Type Constraints for Link Prediction

**DOI:** 10.3390/e24030330

**Published:** 2022-02-25

**Authors:** Xiaohui Li, Zhiliang Wang, Zhaohui Zhang

**Affiliations:** 1 School of Computer & Communication Engineering, University of Science and Technology Beijing, Beijing 100083, China; wzl@ustb.edu.cn; 2 School of Automation and Electrical Engineering, University of Science and Technology Beijing, Beijing 100083, China; zhangzhaohui@ustb.edu.cn

**Keywords:** type constraint, link prediction, complex embedding, complex circular correlation

## Abstract

Large-scale knowledge graphs not only store entities and relations but also provide ontology-based information about them. Type constraints that exist in this information are of great importance for link prediction. In this paper, we proposed a novel complex embedding method, CHolE, in which complex circular correlation was introduced to extend the classic real-valued compositional representation HolE to complex domains, and type constraints were integrated into complex representational embeddings for improving link prediction. The proposed model consisted of two functional components, the type constraint model and the relation learning model, to form type constraints such as modulus constraints and acquire the relatedness between entities accurately by capturing rich interactions in the modulus and phase angles of complex embeddings. Experimental results on benchmark datasets showed that CHolE outperformed previous state-of-the-art methods, and the impartment of type constraints improved its performance on link prediction effectively.

## 1. Introduction

**Figure 1:**
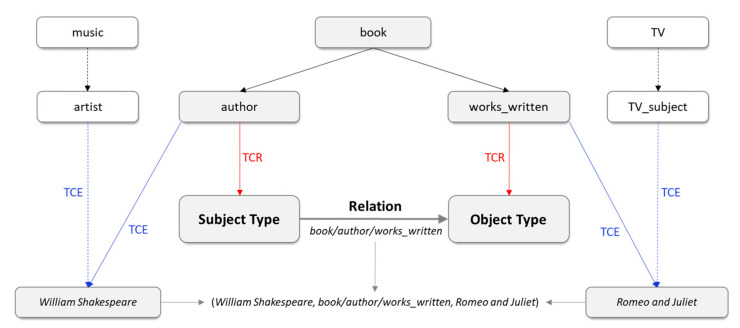
Example of TCE and TCR in Freebase.

Knowledge graphs (KGs), such as Freebase [1], WordNet [2], and YAGO [3], produce massive relational data to support a wide range of applications of artificial intelligence, including recommender systems, question answering, and intelligent search. In these downstream applications of KGs, vast data quantities are organized into directed multigraphs that consist of both knowledge components (entities and concepts) and knowledge structure (relations) [4], and the information processing mechanisms of applications take a knowledge-driven form [5] to make better use the relational data. However, the lack of associations between entities can lead to the incompleteness of knowledge structures, which can directly affect the spread and application of KGs. Hence, the completion of the missing relationships, known as link prediction, has become one of the main problems in relational learning, and all knowledge representation methods prioritize this process. Notably, knowledge graphs form various entities and relationships while also providing a wealth of ontology-based information about them [6]. This information, in particular information about types, can be regarded as abstract semantic constraints and play important roles in knowledge-driven applications. The introduction of type constraints can enhance the accuracy of link prediction and the knowledge discovery ability of KGs, thus improving the integrity of knowledge structures and their practical availability in downstream applications such as question answering systems. Specifically, two types of type constraints—type constraints of entities (TCE, also known as the *instanceOf* relation or entity-type information [7]) and type constraints of relation (TCR)—are crucial for link prediction. Figure 1 shows a triple from Freebase [1] with type constraints to illustrate the roles of TCE and TCR in the structure of knowledge.

In recent years, various knowledge embedding approaches have been proposed and widely used in knowledge graph completion [8], question answering [9] and recommender systems [10] to support specific applications in many industries such as medicine and e-commerce. Most of them encode entities and relationships into low-dimensional real vectors and models to fill in the missing relationships of KGs. Translational models, including TransE [11], TransH [12], and TransR/CTransR [13], utilize distance-based translational properties to handle 1-to-1, 1-to-N, N-to-1, and N-to-N relations. DistMult [14] and RESCAL [15] regard the link prediction task as a 3D binary tensor completion problem [16] and builds relational directed graphs by using the relation matrix or tensor. HolE [17] introduces circular correlation to capture rich interactions between embeddings of entities and implement compositional representations. However, all the above methods ignore the vital role of type information in KGs and only model entities and relationships on the instance view. TKRL [6] investigates the importance of type information for link prediction and uses relation-specific type constraints to achieve outstanding performance. TransC [18] and JOIE [19] jointly model the instance view graph and the ontology view graph of KGs, and illustrate that the introduction of ontology information can improve the performance of link prediction. Unfortunately, most of the existing methods represent the entities, relationships, and types as single real vectors, making it difficult to adequately integrate type constraints into instances and restricting the precision and flexibility of relational learning. More specifically, each element of real-valued vectors is a real number, which only can provide one degree of freedom for modeling the relations between entities. For large-scale KGs, in which the number of dimensions is much smaller than the number of entities and relations, such single degree of freedom representation with real number can cause the position of golden facts to converge on almost one point in the geometric space, which is over-strict for complex relations such as N-to-N relations and type constraints [20].

To address this issue, we sought inspiration from complex representation [16,21] to extend the classic real-valued compositional representation method HolE [17], and proposed a novel complex embedding method named CHolE to represent entities and relationships combined with type constraints. In the proposed approach, entities and the relationship between them were encoded as complex vectors (their types are still embedded as real vectors), which provided two degrees of freedom, modulus and phase angles, for modeling complex relationships and type constraints. Meanwhile, the circular correlation, a real compositional operator proposed by HolE [17], was extended to complex domain and was named complex circular correlation. In complex circular correlation, real-valued multiplication was replaced by complex multiplication, in which the modulus of complex numbers were multiplied and their phase angles performed addition and subtraction. While providing two degrees of freedom for knowledge representation, the multiplication of modulus retained the ability of compositional representation of HolE [17], and the subtraction of phase angles introduced the advantages of distance-based approaches such as TransE [11] to improve the precision and flexibility of relational learning. On the one hand, in CHolE, the real circular correlation [17] and distance-based operation were adopted to model the type constraints and integrated into entities and relationships as the modulus of complex embeddings. On the other hand, the complex version of circular correlation was applied in relational learning to consider the modulus constraints and interactions among phase angles. Correspondingly, CHolE consisted of two main components: the type constraint model (TCM) and the relation learning model (RLM). The TCM, which embeds the types of entities as real vectors in Euclidean space, models the TCE with the traditional distance-based operation similar to TransC [18] and takes the real circular correlation [17] as compositional operator to form the TCR. The TCE and TCR are then imposed on the modulus of complex embeddings to be injected into the entities and relationships. The RLM adopts complex circular correlation to project pairwise interaction in Hermitian dot product [16] of head entity and tail entity on the relationship vector (complex vector) and calculates a sum over a fixed partition [17] of their real parts. In this way, modulus constraints and phase interactions react to type constraints and nonontological interactions, respectively, and are simultaneously integrated by a unified mechanism to capture semantic associations in relationships flexibly. We evaluated our approach on the classic link prediction task, known as entity prediction, and the experimental results showed that CHolE outperformed state-of-the-art methods on benchmark datasets. The contributions of our work can be summarized as follows:

- A novel complex embedding model, named CHolE, was proposed to model relational learning with type constraints, which extended compositional representation HolE [17] to complex domain and injected the type information as modulus constraints into complex embeddings of entities and relations for improving link prediction. It was able to model the entities, relations and the relevant type constraints jointly and effectively utilize their type information for improving link prediction.
- A brand new compositional representation mechanism was developed to integrate the ontology-based information and instance information in KGs. This mechanism used the modulus and phase angles of complex vectors to form the type constraints and nonontological interactions between entities and combined them together with the complex circular correlation to capture multifaceted associations in relations.
- In the experiments, the proposed method outperformed state-of-the-art real-valued knowledge representation methods, including TransE [11], TransH [12], RESCAL [15], DistMult [14], HolE [17], and the classic complex embedding model ComplEx [16], on link prediction tasks. The experimental results on standard benchmark datasets showed that the impartment of type constraints obtained performance gains on link prediction.

The remainder of this paper was organized as follows: Section 2 introduces various methods of knowledge embedding methods for link prediction; Section 3 and Section 4 describe the complex circular correlation, formulation, methodology, and other details of the proposed method. Section 5 reports the dataset, process, and results of our experiments on the proposed model. Section 6 discusses the influence of type constraints on the performance of link prediction, and Section 7 provides the conclusion and future work.

## 2. Related Works

In recent years, various knowledge embedding methods have been proposed, which treat observed facts in KGs as triple sets and can be categorized into three groups: (1) translation-based models (2) tensor factorization-based models, and (3) neural network-based models [22]. In addition to the review of the above three methods, the methods with type information and complex embedding methods were introduced, which were directly relevant to our work.

### 2.1. Translation-Based Models

Inspired by word2vec [23], TransE [11] adopted the scoring function $f_{r}\left(h,t\right)\;=\;\left|\left|h+r-t\right|\right|$ as translation invariant to represent the relationship between entities and introduced a margin-based hinge ranking loss function [11] to improve the performance and effectiveness of the model. A variety of translation-based models, such as TranH [12], TransR [13], and TransD [24], have been proposed successively and extended the original TransE model to address complex relationships, including 1-to-N, N-to-1 and N-to-N. While inheriting the idea of translation invariance of TransE, these models stretch out various relation spaces and project relationships into them to enhance the capacities of knowledge representation. TransH [12] forms relation-specific hyperplanes and projects each entity on them via $e_{⊥}\;=\;e-w^{T}\mathrm{ew}$ to make the same entity produce different embeddings in various relationships. TransR [13] extends relation-specific hyperplanes proposed by TransH [12] to relation-specific spaces and constructs the relation-specific matrix to make projections as $e_{⊥}=M_{r}e$. TransD [24] introduces mapping vectors $w_{h},\;w_{t},\;w_{r}$ to form projection matrix $M_{r}^{h}=w_{r}w_{h}^{T}+I$, $M_{r}^{t}=w_{r}w_{t}^{T}+I$ for head entity and tail entity, respectively, and simplifies the relation-specific matrix in TransR [13].

### 2.2. Tensor Factorization-Based Models

Such methods regard link prediction as a 3D binary tensor completion problem [16] and encode relational directed graph by using the relational matrix or tensor. The core idea of tensor factorization is to map all relationships in KGs into 3D tensor structure $X\in {\mathbb{R}}^{n\times n\times m}$, and each entry $x_{ijk}$ of tensor indicates whether a *k*-th type of relationship is found between the *i*-th entity and the *j*-th entity. RESCAL [15] introduced a bilinear model to obtain the latent semantic associations between entities and calculate the scoring function $f\left(h,r,t\right)=h^{T}M_{r}t$ with relation matrix $M_{r}$ to determine the existence of relationships. DistMult [14] restricts the relation matrix into diagonal matrices to simplify the computational complexity of RESCAL. HOLE [17] introduced circular correlation as a compositional operator, which can be interpreted as compression of tensor products of the head and tail entities to capture pairwise interactions of entity features [17] and equivalently utilizes fast Fourier transform (FFT) [25] to accelerate its computational process. A few complex tensor factorization methods, introduced in a separate paragraph later in this work, have been developed and made progress in performance.

### 2.3. Neural Network-Based Models

Neural networks, especially deep networks, have powerful capabilities of complicated relational learning and are widely applied in knowledge representation and link prediction. SME [26] introduced linear and bilinear networks to calculate energy functions, which can be used to measure the confidence of semantic relation matching. NTN [27] applies a neural tensor network, which replaces standard linear layers with bilinear tensor layers to depict complicated semantic relations more precisely. ConvKB [28] introduced a convolutional neural network to generate feature maps of triples and capture the latent semantic relations with them. R-GCN [29] improved graph neural network for knowledge graph representation, which provides relation-specific weight matrices to identify various relationships between an entity and its neighbors. The multi-scale dynamic convolutional network (M-DCN) [30] generates multi-scale convolution filters in the convolution layer to learn different characteristics among input embeddings for modeling the complex relations in KGs. HyperGEL [31] extends hyperbolic graph neural network by introducing the relation features and forms an encoder–decoder hyperbolic embedding learning framework for KG completion.

### 2.4. Methods with Type Information

In addition to entities and relationships, most KGs contain tremendous type information, which plays an important role in link prediction. In recent years, some embedding methods with type information have been proposed, which either integrate type information into relational learning or focus on the joint representation of the ontology and instance views of KGs. TKRL [6] constructs type-specific projection matrices $M_{rh},{\;M}_{rt}$ for head entity and tail entity and defines the energy function $E\left(h,r,t\right)=\left|\left|M_{rh}h+r-M_{rt}t\right|\right|$ to capture multiple-category semantic information in entities for enhancing the embedding model. TransC [18] embeds types as spheres in Euclidean space and uses the geometric inclusion to depict the hierarchy structure and the instantiation of types. JOIE [19] proposed a multi-view embedding framework, which composed of the ontology view and the instance view, and establishes intra-view component and cross-view component to model hierarchy-aware structure of types and their instantiations.

### 2.5. Complex Embedding Methods

Recently, increasingly complex embedding methods have emerged and demonstrated their strong representation capabilities in KGs. ComplEx [16] first introduced complex embedding into the domain of knowledge representation and used the Hermitian dot product to extend DistMult [14] to complex vector space so as to address asymmetric relations more effectively. RotatE [21] embeds the entities and relations to the complex vector space and defines each relation as a rotation from the head entity to the tail entity, which can effectively model various relation patterns, including inversion, symmetric/antisymmetric, and composition. QuatE [32] further extends complex space into 4D hypercomplex space known as quaternion space and adopts the Hamilton product to capture richer latent semantics meanings in entities and construct more compact interaction structure between them. DualQuatE [33] introduces dual quaternion into knowledge graph embedding and uses both rotation and translation simultaneously to represent various relations between entities in KGs.

## 3. Preliminaries

In this section, complex circular correlation, the complex version of circular correlation [17], was briefly introduced and used as compositional operator in the proposed model. The problem of relational learning with type constrains involved in our method and experiment is formulated in detail.

### 3.1. Complex Circular Correlation

#### 3.1.1. HolE and Circular Correlation

Holographic embedding (HolE) [17] is one of the most remarkable compositional representation methods, which is related to holographic models of associative memory in that it introduces circular correlation as compositional operator to create binary relational representations. Plate [34] investigated circular correlation, circular convolution and aperiodic convolution as compressed outer products of two vectors for forming associations in holographic reduced representations. In HolE [17], circular correlation, which calculates a sum over a fixed partition of pairwise interactions in tensor product, was similarly employed as a compression of the tensor product to capture rich interactions while simultaneously making the model concise and efficient [17]. Concretely, ℝ denotes the sets of real values, and $a,\;b\in {\mathbb{R}}^{n}$ denote two *n*-dimensional real vectors. The circular correlation ⋆: ${\mathbb{R}}^{n}\times {\mathbb{R}}^{n}\to {\mathbb{R}}^{n}$ is defined [17] as

(1) $${\left[a⋆b\right]}_{j}=\overset{n-1}{\underset{k=0}{\sum }}a_{k}b_{\left[\left(j+k\right)\;mod\;n\right]},$$

**Figure 2:**
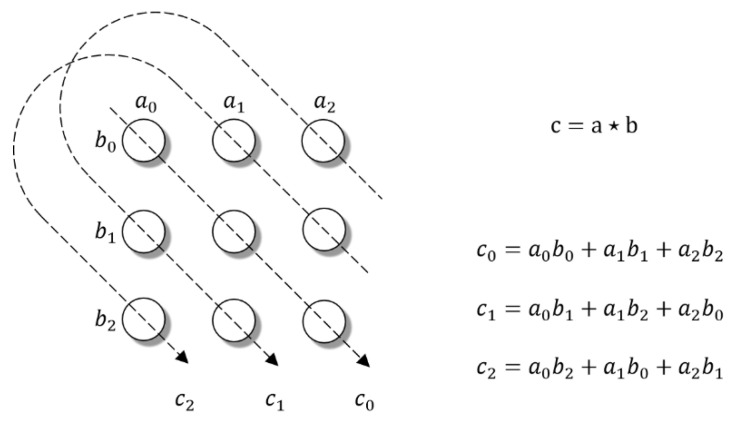
Circular correlation as the compression of the tensor product.

In Equation (1), the circular correlation compresses the real tensor product by summing over the interactions of tensor product in accordance with the subscript rule $sr\left(j,k\right)=\left(j+k\right)\;mod\;n$, as illustrated in Figure 2 [34].

In the relation-specific scoring function $\sigma (r_{p}^{T}\left(a⋆b\right))$, where $r_{p}$ denotes the real vector of the *p*-th type relation and $\sigma \left(\cdot \right)$ is the logistic function, all $n^{2}$ interactions in tensor product matrices are grouped into n partitions by subscript rule, which are summed up separately. In the above scoring function, the relation vector assigns the weights for each partition to separate the possible interactions relevant to relation-specific pattern from the irrelevant interactions [17]. Although circular correlation provides strong semantic interaction capabilities, it does not increase the dimensionality of the composite representation [17]. Its computational process can be accelerated via FFT as follows:

(2) $$a⋆b=F^{-1}\left(\bar{F(a)}⊙F\left(b\right)\right),$$

where $F\left(\cdot \right)$ and $F^{-1}\left(\cdot \right)$ denote the FFT and its inverse, $\bar{x}$ denotes the complex conjugate of x∈ℂ, and ⊙ denotes the Hadamard product.

#### 3.1.2. Complex Circular Correlation

In this work, a novel complex compositional operator named complex circular correlation was introduced. This operator extended the real circular correlation to complex domains to model the entities and relationships with ontology constraints. With regard to complex vectors $u,\;v\;\in {\mathbb{C}}^{n}$, the tensor product in real circular correlation was expanded to the Hermitian product, which is defined as

(3) $$\langle u,v\rangle ={\bar{u}}^{T}v,$$

where $\bar{x}$ denotes the complex conjugate of $x\in {\mathbb{C}}^{n}$, and an entry in it can be denoted as

(4) wjk=u¯jvk=Reuj−i·Imuj⋅Revk+i·Imvk,

where $u_{j},\;v_{k}\in \mathbb{C}$ are complex numbers, Re⋅ and Im⋅ denote the real part and the imaginary part of a complex number, respectively, and ${\bar{u}}_{j}$ denotes the conjugate of $u_{j}\in \mathbb{C}$. We rewrote Equation (4) in a form that corresponded with the polar coordinates to divide operations into dot product and phase rotation, as follows:

(5) $$\begin{array}{l} w_{jk}={\bar{u}}_{j}v_{k}\\ =\left(m\left(u_{j}\right)\cdot e^{-i\cdot \theta (u_{j})}\right)\cdot \left(m\left(v_{k}\right)\cdot e^{i\cdot \theta (v_{k})}\right)\\ =m\left(u_{j}\right)m\left(v_{k}\right)\cdot e^{i\cdot \left(\theta (v_{k}\right)-\theta (u_{j}))}, \end{array}$$

where i is the imaginary unit, $m\left(\cdot \right)\;$denotes the moduli of complex numbers, and $\theta \left(\cdot \right)$ returns its phase angle in the range $\left[-\pi ,\pi \right]$. In this form, the squared moduli mx2=Rex2+Imx2 can be interpreted as the energy of a complex number, and be assigned into the real and imaginary parts by Rex=mxcosθx,Imx=mxsinθx. If $m{\left(x\right)}^{2}$ is on, then its components Rex2,Imx2 have the possibility of being on, which depends on the phase angle $\theta \left(x\right)$. For the entry $w_{jk}$ in Hermitian product 〈u,v〉, the moduli $m\left(u_{j}\right),m\left(v_{k}\right)$ is distributed to the real and imaginary parts in accordance with their difference in phase angle $\theta (v_{k})-\theta (u_{j})$. 

**Figure 3:**
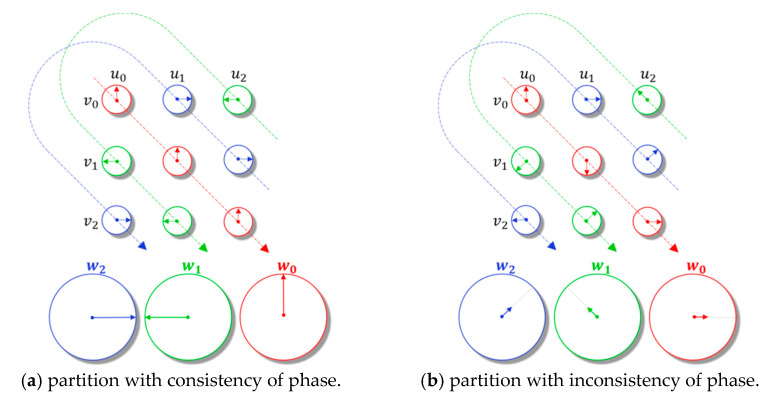
Summation processes over a fixed partition in the complex circular correlation.

The Hermitian product 〈u,v〉 was partitioned, and the interactions in a partition were summed up in a similar manner as real circular correlation [17] to define the complex circular correlation as

(6) $${\left[u⋆v\right]}_{j}=\overset{n-1}{\underset{k=0}{\sum }}{\bar{u}}_{k}v_{\left[sr\left(j,k\right)\right]}=\overset{n-1}{\underset{k=0}{\sum }}m\left(u_{k}\right)m\left(v_{\left[sr\left(j,k\right)\right]}\right)\cdot e^{i\cdot \left(\theta \left(v_{\left[sr\left(j,k\right)\right]}\right)-\theta \left(u_{k}\right)\right)},$$

where $u,v\in {\mathbb{C}}^{n}$ are the complex vectors, and ${\left[u⋆v\right]}_{j}$ is the *j*-th entry of u⋆v. In Equation (6), the polar form of complex circular correlation is provided, where $m\left(\cdot \right),\;\theta \left(\cdot \right)$ denote the moduli and phase angle of complex number, respectively, and $sr\left(j,k\right)=\left(j+k\right)\;mod\;n$. Assuming that the moduli of ${\bar{u}}_{k}$ and $v_{\left[sr\left(j,k\right)\right]}$ are fixed, if and only if the phase angles of all interactions in a partition are the same (with consistency of phase), ideally, then the moduli of the sum over them take the maximum value. Figure 3 illustrates the sum over a fixed partition with consistency of phase angles and inconsistency of phase angles.

The complex circular correlation can be accelerated via FFT similar to its real version [17]. Specifically, it can be divided into four parts by following the different combinations of real and imaginary parts as

(7) u⋆v=Reu⋆Rev+Imu⋆Imv+i·Reu⋆Imv−Imu⋆Rev,

where ⋆ on the left-hand side of the equation denotes the complex circular correlation operator, and ⋆ on the right-hand side represents its real version. Thus, the complex circular correlation can be obtained by calculating the FFT of four components in Equation (7) and taking their algebraic sum.

#### 3.1.3. Mechanisms of Modulus Constraint and Phase Interaction

With the complex circular correlation, the corresponding scoring function is introduced to form the modulus constraints and the phase interaction. The scoring function similar to HolE [17] is defined as

(8) fh,r,t=σ(Re(r¯Th⋆t)),

where h and t are complex vectors of the head and tail entity, respectively, $\bar{r}$ is the conjugate of complex vector of the relationship in triple $\left(h,r,t\right)$, ⋆ denotes the complex circular correlation in Equation (5), Re⋅ takes the real part of complex number, and $\sigma \left(\cdot \right)$ denotes the logistic function. We investigate the *j*-th interaction of Re(r¯Th⋆t) in Equation (8) as follows:

(9) Rer¯Th⋆tj=Rerj¯∑k=0n−1h¯ktsrj,k=Remrjei⋅−θrj∑k=0n−1mhkmtsrj,kei⋅θtsrj,k−θhk=mrj∑k=0n−1mhkmtsrj,k·Re(ei⋅θtsrj,k−θhk−θrj),

where $r_{j},h_{k},t_{\left[sr\left(j,k\right)\right]}$ denotes the corresponding entries of r,h,t**,** respectively, and all other symbols have the same meaning as in Equations (6) and (8).

**Figure 4:**
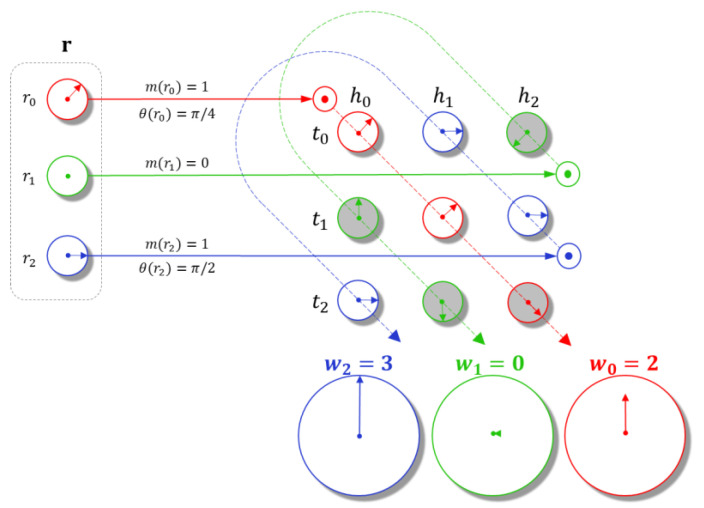
Mechanisms of Modulus Constraint and Phase Interaction.

In Equation (9), the phase interaction is described as Re(ei⋅θtsrj,k−θhk−θrj). It is defined as the real part of unit-length complex number, and its phase angle is equal to $\theta \left(t_{\left[sr\left(j,k\right)\right]}\right)-\theta \left(h_{k}\right)-\theta \left(r_{j}\right)$. When the difference between the $t_{\left[sr\left(j,k\right)\right]}$ and $h_{k}$ satisfies $\theta \left(t_{\left[sr\left(j,k\right)\right]}\right)-\theta \left(h_{k}\right)=\theta \left(r_{j}\right)$, the value of phase interaction is equal to one. If $\theta \left(t_{\left[sr\left(j,k\right)\right]}\right)-\theta \left(h_{k}\right)-\theta \left(r_{j}\right)=\pm \;\pi /2$, then it takes on the value zero. Although the three phase angles of $t_{\left[sr\left(j,k\right)\right]},\;\;h_{k},\;\;r_{j}$ take other values, the value of the phase interaction varies in the range $\left[-1,\;1\right]$. The modulus constraint is defined as follows: let all the phase interactions be one, then Rer¯Th⋆tj in Equation (9) becomes the modulus constraint and degrades into a real circular correlation, where the values of entries of the relation vector and all interactions are nonnegative. In an overall view, the entries of relation vector r in Equation (9) can pick out the interactions with consistency of phase angles and make them available in complex circular correlation. In other words, the modulus constraint limits the energy of each interaction, and the phase interaction determines the weight of its energy projection on the real part. Figure 4 illustrates the modulus constraint and phase interaction in detail.

In the proposed model, the modulus constraint was used to represent the type constraints of entities and relations, and the interactions between entities other than type constraints were modeled with the phase interaction, as elaborated in Section 4.

### 3.2. Problem Formulation

The problem of integrated embedding of KGs with type constraints was formulated in complex space, which consisted of entities and various relationships between them and their type constraints. For a clear illustration, Table 1 gives a summary of all symbols used in this paper.

Given a knowledge graph that includes entities, types, and various relations, it can be formalized as KG = {E,C,R,S}, where E is the entity set, C is the type set (also known as concept set), R is the relation sets, and S is the triple set, to denote the relational facts. In this formulation, relation sets R *=* R*_I_*∪R*_TC_* consist of two subsets, R*_I_* and R*_TC_*, which denote the instance-level relation and the type constraint relation, respectively. Similarly, the triple sets S *=* S*_I_*∪S*_TC_* are divided into two subsets, S*_I_* and S*_TC_*, to denote the relational facts of instance-level relation and its type constraints.

Here, the specific relations between entities, which are called instance-level relation, are distinguished from the type constraints: (1) Instance-level relation, which is denoted as $r_{j}\in$R*_I_*, indicates the relation between entities. For example, the “*writtenBy*” is an instance-level relation and can connect the entity “*Shakespeare*” and “*Romeo and Juliet*” directly to represent the fact “*Romeo and Juliet is written by Shakespeare*”; (2) Type constraint relation, which indicates the TCE (also known as the *instanceOf* relation) and the TCR, is denoted as R*_TC_ =* {$r_{TCE}$,$r_{TCR}$}. For the relation of TCE $r_{TCE}$, each entity e∈ E belongs to at least one type c∈C, and one or more instances of a type are found in KG. For example, the entity “*Shakespeare*” is the instantiation of one type “*author*”, and another type “*written_work*” has an instance “*Romeo and Juliet*”. The relation of TCR $r_{TCR}$ indicates the types of head and tail entity for an instance-level relation. For instance, the “*writtenBy*” relation has the head type “*written_work*” and the tail type “*author*”. In each fact by this relation $\left(h,r,t\right)$, the head entity belongs to the head type and this is the same with the tail entity and the tail type. Two types of triple sets were used to denote the facts of the Instance-level relation and type constraints, including the TCE and TCR: (1) General triple set, which contains a mass of facts of various instance-level relations, is formalized as S*_I_* $=\{\left(h,r_{j},t\right)|h,t\in$ E $\mathrm{and}\;r_{j}\in$R*_I_* }; (2) Type constraint triple sets can be divided into the TCE triple set and TCR triple set. The TCE triple set is formalized as S*_TCE_* $=\{\left(c,r_{TCE},e\right)|e\in$ E and c∈C} because the entity e is one of instantiations of the type (concept) c. TCR triple set is defined as S*_TCR_* $=\{\left(c_{h},r_{j},c_{t}\right)|c_{h},c_{t}\in$C $\mathrm{and}\;r_{j}\in$R*_I_*}, where $c_{h}$ and $c_{t}$ denote the head and tail type of the relation $r_{j}$, respectively. 

In the proposed method, the entity e∈ E and instance-level relation $r_{j}\in$R*_I_* were embedded into complex vector space. Specifically, the entity *e* was mapped to an *n*-dimensional complex vector $e\in {\mathbb{C}}^{n}$, and Ree, Ime, me, and θe∈ℝn are four *n*-dimensional real vectors that encoded the real part, imaginary part, modulus, and phase angle of the complex vector e, respectively. Likewise, the instance-level relation $r_{j}\in$R*_I_* is represented as complex vector $r_{j}$, and Rerj, Imrj, mrj, and θrj∈ℝn are defined similarly. We still embedded the types of entities c∈ C to real vector space as *n*-dimensional real vector $c\in {\mathbb{R}}^{n}$.

## 4. Methodology

### 4.1. Overview

**Figure 5:**
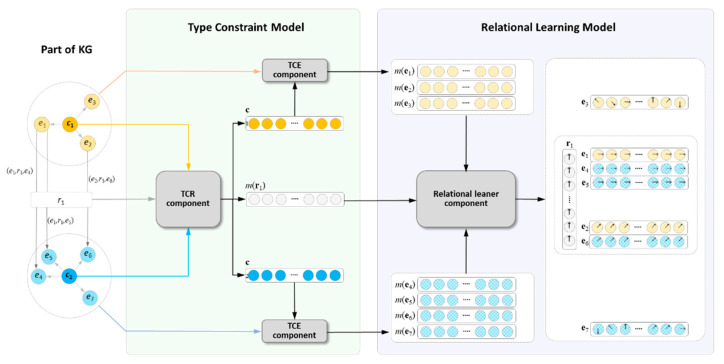
An overview of CHolE model. The leftmost part contains a part of KG that includes seven entities, their types, and one relationship between them. The TCM learns the TCE and TCR with the modulus of complex vectors (green box). The RLM models the detailed interactions with the modulus and phase angles of complex (blue box). The solid circle denotes the real number, and the slash-marked circle with arrow line denotes the complex number (slash-marked circle without arrow line is the moduli of complex number). For a brief description: let the relationship of phase angles be zero and simplify the phase angles of golden entity pairs to be the same to make the phase difference of entries in head and tail entities be zero (this can be also regarded as a special case in Equation (9).

In this section, the complex embedding method CHolE was introduced in detail. CHolE focused on relational learning with type constraints and addressed the link prediction problem more accurately by using the type information of entities and relations. In the proposed approach, the entities and relations were embedded as *n*-dimensional complex vectors, and their modulus vectors and phase angle vectors were used to capture the type constraints and interactions between entities other than type constraints. As shown in Figure 5, CHolE was composed of two main functional parts: the TCM and the RLM. The TCM had two key components, namely: TCE and TCR. These dealt with the TCE and TCR, respectively, with the modulus of complex vectors. The RLM integrated the type constraints formed by TCE and TCR components and adopted complex circular correlation as compositional operator to learn more detailed interactions between entities with the phase interactions. These models and components are detailed in Figure 5.

### 4.2. TCM

The goal of TCM is to represent the type constraints in KGs, including the TCE and TCR. The TCE establishes a correspondence between an entity and its type (also known as the *instanceOf* relation), and the TCR limits the types of head entity and tail entity in certain relationships. Accordingly, two components of TCM, the TCE component and TCR component, were developed to address the two types of constraints with the modulus of complex vectors. In CHolE, an entity was embedded as an *n*-dimensional complex vector $e\in {\mathbb{C}}^{n}$, and its modulus vector and phase angle vector were encoded as $m\left(e\right),\;\theta \left(e\right)\in {\mathbb{R}}^{n}$. Likewise, the relationship between entities was formalized as complex vector $r\in {\mathbb{C}}^{n}$ and defined as $m\left(r\right),\;\theta \left(r\right)\in {\mathbb{R}}^{n}$. The type of entities (also known as concept) was encoded as a real vector $c\in {\mathbb{R}}^{n}$.

#### 4.2.1. TCE Component

The TCE constraint between entity and its type, otherwise known as *instanceOf* relation, is considered a basic ontology constraint and exists widely in KGs. In the TCE component, we adopted the distance-based scoring function proposed by TransC [18] to model the *instanceOf* relation. The distance range of types varied depending on the number of entities that the type contained. Concretely, given a complex vector of an entity $e\in {\mathbb{C}}^{n}$ and a real vector of its type $c\in {\mathbb{R}}^{n}$, we defined the scoring function to measure the existence of *instanceOf* relation as follows:

(10) $$f_{TCE}\left(c,e\right)=\mathrm{Relu}\left({∥m\left(e\right)-m\left(c\right)∥}_{2}-\frac{num_{c}}{num_{E}}\cdot br\right),$$

where $e\in {\mathbb{C}}^{n},\;c\in {\mathbb{R}}^{n}\;$ are the embedding vectors of entity and type, $m\left(\cdot \right)\in {\mathbb{R}}^{n}$ is the modulus vector of complex vector and real vector, $∥m\left(e\right)-m\left(c\right){∥}_{2}$ is the dissimilarity measure by the L2-norm between $m\left(e\right)$ and $m\left(c\right)$, $\mathrm{Relu}\left(\cdot \right)$ signifies the ReLU activation function, and $num_{c}∕num_{E}\cdot br$ indicates the threshold of distance between $m\left(e\right)$ and $m\left(c\right)$. Here, br≥0 is a hyperparameter that denotes the base of range (radius) for types, $num_{c}\in {\mathbb{N}}^{+}$ denotes the quantity of entities belonging to type c, and $num_{E}\in {\mathbb{N}}^{+}$ is the total amount of e∈ E. Thus, the entities of a certain type were located in a sphere [18] in Euclidean space, where its center was the endpoint of real vector $m\left(c\right)$, and its radius depended on the proportion of the number of entities it owned in the total in KG. If one type has numerous entities, these entities are supposed to be distributed into spheres with larger radii to ensure that each entity is distinguishable. If only a few entities belong to a type, then the smaller radius is obtained using Equation (10) to emphasize the similarity among them, while ensuring differentiation.

A margin-based hinge loss function was minimized to learn the *instanceOf* relations and discriminate positive triples from others, which can be expressed as follows:

(11) $$L_{TCE}^{S_{TCE}}=\frac{1}{{|S}_{TCE}|}\sum_{\xi \in S_{TCE}\;}\;\sum_{\xi^{\prime }\notin S_{TCE}}{\left[\gamma_{TCE}+f_{TCE}\left(\xi \right)-f_{TCE}\left(\xi^{\prime }\right)\right]}_{+}\;,$$

where $S_{TCE}$ denotes the set of correct triples, and ξ is a positive triple $\left(c,r_{TCE},e\right)$, $\xi^{\prime }$ denotes negative triple $\left(c,r_{TCE},e^{\prime }\right)$ or $\left(c^{\prime },r_{TCE},e\right)$ by corrupting ξ, ${\left[x\right]}_{+}=\max\left(0,x\right)$ and $\gamma_{TCE}>0$ is the margin to separate positive triples and negative triples. For an *instanceOf* triple $\left(c,r_{TCE},e\right)\in S_{TCE}$, we stochastically replace type c with alternatives $c^{\prime }\in \{c_{j}|c_{j}\in C\;\mathrm{and}\;c_{j}\neq c\}$ or replace entity e with $e^{\prime }\in \{e_{j}|e_{j}\in E\;\mathrm{and}\;e_{j}\neq e\}$ and filter those candidates in $S_{TCE}$.

#### 4.2.2. TCR Component

Similar to the TCE, type constraints, which are called TCR, exist in various relations. For a specific relationship $r\in R_{I}$, its head entity and tail entity are restricted to specific types. TKRL [6] used type-specific projection to form h and t and constrained them to specific types $c_{rh},c_{rt}$, where head and tail should belong in this relation. In the proposed model, the TCR component was established with the modulus of complex vector for relationship r, and the circular correlation was used as compositional operator to model the type constraint of relation. We defined the TCR scoring function similar to HolE [17] as

(12) $$f_{TCR}\left(c_{rh},r,c_{rt}\right)=\sigma \left(m{\left(r\right)}^{T}\left(c_{rh}⋆c_{rt}\right)\right),$$

where $c_{rh},{\;c}_{rt}\in {\mathbb{R}}^{n}$ are the real vector representations of relation-specific types, $m\left(r\right)$ denotes the modulus vector of complex vector $r\in {\mathbb{C}}^{n}$, $x^{T}$ indicates the transposition of vector x, ⋆ denotes the real circular correlation defined in Equation (1), and $\sigma \left(x\right)=1∕\left(1+e^{-x}\right)$ is the logistic function. The hinge loss function was applied and minimized for training of the TCR component:

(13) $$L_{TCR}^{S_{TCR}}=\frac{1}{{|S}_{TCR}|}\sum_{\tau \in S_{TCR}\;}\;\sum_{\tau^{\prime }\notin S_{TCR}}{\left[\gamma_{TCR}+f_{TCR}\left(\tau^{\prime }\right)-f_{TCR}\left(\tau \right)\right]}_{+},$$

where τ is the positive triple $\left(c_{rh},r_{TCR},c_{rt}\right)\in S_{TCR}$, $\tau^{\prime }$ denotes the corrupted triple $\left(c_{rh}{}^{’},r_{TCR},c_{rt}\right)$ or $\left(c_{rh},r_{TCR},c_{rt}{}^{’}\right)$, the positive margin of hinge function is denoted as $\gamma_{TCE}$, and ${\left[x\right]}_{+}=\max\left(0,x\right)$. We corrupted the positive triple by randomly replacing type $c_{rh}\;\mathrm{or}\;c_{rt}$ with other candidates $c^{\prime }\in \{c_{j}|c_{j}\in C,\;\mathrm{and}\;c_{j}\neq c_{rh}\;\mathrm{and}\;c_{j}\neq c_{rt}\}$.

In summary, we defined the complete loss function for the whole TCM as

(14) $$L_{TC}^{S_{TC}}=L_{TCE}^{S_{TCE}}+L_{TCR}^{S_{TCR}},$$

Thus, the type constraints, which represent entities and relationships, are marked on the modulus of complex vectors and make them satisfy the TCE and TCR simultaneously.

### 4.3. RLM

As mentioned previously, the mechanism of TCM, which generates relation-specific types and groups entities into them with modulus of complex vectors, was described. In this subsection, the RLM was introduced to capture more specific semantic relatedness between each entity pair with phase interactions and learn various instance-level relationships by complex circular correlation. In the RLM, entities and relationships between them were embedded as complex vectors to combine phase interactions with modulus constraints that provide type information about them. The mechanism of complex circular correlation with modulus constraint and phase interaction was described in detail in Section 3.1. Here, we defined the scoring function and the loss function for RLM directly. Given head entity h∈E, tail entity t∈E and a certain relationship $r\in R_{I}$, their complex vector representations were $h,\;t,\;r\in {\mathbb{C}}^{n}$, respectively. The scoring function of relational learning is defined as

(15) fRLh,r,t=σ(Re(r¯Th⋆t)),

where $\bar{x}$ indicates the conjugate of complex vector x, ⋆ denotes the complex circular correlation described in Equation (5), Re⋅ denotes the real part of complex number and $\sigma \left(\cdot \right)$ is the logistic function. The corresponding hinge loss function was defined and minimized as

(16) $$L_{RL}^{S_{I}}=\frac{1}{{|S}_{I}|}\sum_{\xi \in S_{I}\;}\;\sum_{\xi^{\prime }\notin S_{I}}{\left[\gamma_{LR}+f_{LR}\left(\delta^{\prime }\right)-f_{LR}\left(\delta \right)\right]}_{+},$$

where δ denotes positive triple $\left(h,r,t\right)\in S_{I}$, $\delta^{\prime }$ denotes negative triple $\left(h^{\prime },r,t\right)$ or $\left(h,r,t^{\prime }\right)$, $\gamma_{RL}>0$ is the margin to distinguish between positive and negative examples and ${\left[x\right]}_{+}=\max\left(0,x\right)$. In negative sampling, for a relational triple$\;\left(h,r,t\right)$, the alternative entity is randomly picked up from the entity set $\bar{E}=\{e_{j}|e_{j}\in E\;,\;\mathrm{and}\;e_{j}\neq h\;\mathrm{and}\;e_{j}\neq t\}$. The bern strategy was adopted, as discussed in Wang et al., 2014 [12], to calculate different probabilities for replacing the head or tail entity.

We integrated $L_{TC}^{S_{TC}}$ and $L_{RL}^{S_{I}}$ and defined the overall loss function as their weighted sums

(17) $$L=L_{LR}^{S_{I}}+\alpha \cdot L_{TC}^{S_{TC}},$$

where $\alpha \in \left[0,1\right]$ is the hyperparameter as the balance factor to leverage two loss functions $L_{TC}^{S_{TC}}$ and $L_{RL}^{S_{I}}$.

## 5. Experiments

In this section, we presented our experiments and evaluation results of CHolE on link prediction. To evaluate the performance of our approach, we first described the benchmark datasets used in our experiments. We then introduced the evaluation protocol, baselines, and implementation details of our experiments. The experimental results showed that the proposed model outperformed state-of-the-art baselines.

### 5.1. Datasets

In this work, we evaluated our approach on two benchmark datasets: FB15K-571 [6] and FB15K-237-TC. The vast majority of previous studies used FB15K [11], WN18 [11] and their variants for model evaluation. FB15K-571 is a variant of FB15K developed by Xie et al. [6] for evaluating the performance of KG embedding with type information. It contains 571 types of entities, 123,842 TCE (*instanceOf*) triples, and the relation-specific type information (TCR information) of 1345 relationships [6]. To avoid the inverse relation loophole in evaluation [35], we constructed a new dataset named FB15K-237-TC from FB15K-237 [35], which was widely applied in knowledge embedding and removed inverse relations in a similar manner as FB15K-571: collecting types of entities through the *type/instance* field and the relation-specific type information located in *rdf-schema#range* field in FB15K [6]. The detailed statistics of FB15K-571 and FB15K-237-TC are shown in Table 2.

### 5.2. Experiment Settings

Link prediction is a common task for knowledge graph evaluation, and its goal is to predict the missing entities or relations in incomplete triples. Following existing studies about knowledge embedding [11,12,13,16], the link prediction was implemented with our approach in the scenario below: given the partial triple $\left(?,r_{I},e_{t}\right)$ or $\left(e_{h},r_{I},?\right)$, the proposed model was trained with triples in the training set, and the trained embeddings were used to predict the missing head entity or tail entity. In our experiment, we performed link prediction on two datasets and compared it with baseline models.

#### 5.2.1. Baselines

To compare the performance of our model in link prediction, we elected six representative state-of-the-art models as baselines that included translation-based models (TransE [11], TransH [12]), tensor factorization-based models (RESCAL [15], DistMult [14], HolE [17]), and the classical complex embedding method (ComplEx [16]). Following most relational learning methods with ontology information [6,18,19], the pairwise ranking loss [11] was used for evaluation. In the FB15K-571 dataset, we implemented ComplEx with pairwise ranking loss for comparison and trained the method using the recommended parameters provided by the authors [16].

#### 5.2.2. Evaluation Protocol

For evaluation, we used the same ranking method proposed by TransE [11]. Concretely, for each triple $\left(h,r,t\right)$ in the test set, either head entity h or tail entity t was removed and replaced by all other members in the entity set to generate candidate triples $\left(h^{\prime },r,t\right)$ and $\left(h,r,t^{\prime }\right)$. The scores were calculated using function $f_{RL}$ in Equation (15) and ranked in “Raw” and “Filtered” settings [12]. In the “Raw” setting, all the restructured triples were ranked, and in the “Filtered” setting, those triples that existed in the training, validation or test set were removed from the list of candidates. Following previous studies, we adopted two standard evaluation metrics on the link prediction to compare the performance of our model with the baselines: (1) the mean reciprocal rank (MRR) of all correct triples, (2) the proportion of positive triples in the test set ranked in top 1, top 3, and top 10 (as Hits@1, Hits@3, Hits@10). All the metrics were positive indicators where higher value was expected and implied better performance of models.

#### 5.2.3. Implementation Details

In the experiment, we implemented CHolE with Tensorflow2.6 and ran it on the host equipped with NVIDIA RTX 3060 graphics processing units. Two versions of our model, the CHolE (only RL) and the CHolE (TC+RL), were provided to evaluate the improvement of type constraints on link prediction. The “only RL” version contained only the RLM but not TCM, and the “TC+RL” version covered the two main components. In the training, we adopted the stochastic gradient descent (SGD) [36] algorithm to minimize the loss function and utilized Adam [37] as the optimizer to find the best hyperparameters for updating embedding on the validation set. We trained our model until convergence but stopped, at most, at 1500 rounds. For the hyperparameters, we selected the dimensionality n among$\;\left\{50,\;100,\;150,\;200,\;250,\;300,\;350,\;400\right\}$, the learning rate lr for SGD among $\left\{0.0001,\;0.0005,\;0.001,\;0.005,\;0.01,\;0.1\right\}$, the margins of hinge loss $\gamma_{TCE},\gamma_{TCR},\gamma_{LR}$ among $\left\{0.1,\;0.15,\;0.2,\;0.3,\;0.5,\;0.7,\;1.0\right\}$, the base of type radius br in Equation (10) among $\left\{0,\;0.25,\;0.5,\;0.75,\;1.0,\;1.25,\;1.5,\;1.75,\;2.0\right\}$, the balance factor of losses α in Equation (17) among $\left\{0,\;0.1,\;0.2,\;0.3,\;0.4,\;0.5,\;0.6,\;0.7,\;0.8,\;0.9,\;1.0\right\}$, the batch number per epoch for general relation triples, and *instanceOf* triples among $\left\{100,200,500,1000\right\}$. Negative sampling was performed on TCM and RLM with a negative sampling ratio 1 in the training process, and the Xavier initializer [38] was deployed to keep the scale of the initial embeddings constant.

### 5.3. Results of Link Prediction

Table 3 shows the evaluation results on FB15K-571 and FB15K-237-TC datasets for link prediction. The two versions (“only RL” and “TC+RL”) of CHolE were compared with the baselines listed below. From the results shown in Table 3, we observed the following:

- CHolE outperformed baseline models on most of the metrics for link prediction on FB15K-571 and FB15K-237-TC. This condition demonstrated that the proposed complex embedding method was effective and promising, and the impartment of type constraints considerably improved the performance on link prediction.
- Compared with the original HolE [17], the experimental results of the “RL only” version of CHolE were higher on FB15K-237-TC, but most of the metrics, including MRR (Filtered), Hits@1, Hits@3, and Hits@10 were slightly lower than HolE [17], and the MRR (Raw) was flat on FB15K-571. This finding was partially because the complex circular correlation in CHolE led to more complicated and rigorous constraints with modulus and phase angles, which were more difficult to reach. However, with the introduction of type constraints, the entities were grouped into their relation-specific types with modulus to make the modulus constraint harder, and the greater possibility of phase matching was obtained. Most of the experimental results indicated that the full version (“TC + RL”) of CHolE performed better than HolE [17] on two datasets. In the FB15K-571 dataset, CHolE (TC+RL) obtained 0.019 higher MRR (Filtered), 2.2% higher Hits@1, 2.4% higher Hits@3 and 0.7% higher Hits@10. In the FB15K-237-TC dataset, the full version of CHolE obtained 0.061 higher MRR (Raw), 0.059 higher MRR (Filtered), 7% higher Hits@1, 5.8% higher Hits@3 and 5.7% higher Hits@10.
- Compared with the complex embedding ComplEx [16], the “RL only” version of CHolE obtained higher results on most metrics, and the “TC+RL” version made significant progress on two datasets. As seen in Table 3, CHolE(TC+RL) obtained 0.058 higher MRR (Filtered), 7.7% higher Hits@1, 6% higher Hits@3 and 1.7% higher Hits@10 on FB15K-571, and 0.08 higher MRR (Filtered), 9.1% higher Hits@1, 9.8% higher Hits@3 and 6% higher Hits@10 on FB15K-237-TC. We ascribed the improvement of the full version of CHolE to having utilized the modulus and phase angles to capture the semantic relatedness on ontology and instance view, respectively. By contrast, the ComplEx [16] extended DistMult [14] to complex space. It neither took full advantage of the modulus and phase angles of complex representational vectors nor integrated type constraints into relational interactions with them.

## 6. Discussion

In this subsection, we examined how the critical parameters of TCM affected the performance on link prediction and investigated the balance factor of losses α and the base of type radius br. This process was performed to study the effects of type constraints on link prediction. The balance factor of losses, denoted as α in Equation (17), was used for leveraging two losses $L_{TC}^{S_{TC}}$ and $L_{RL}^{S_{I}}$. The base of type radius br in Equation (10) indicated the radius scale of types and was used to calculate the radius value combined with the proportion of type-specific instances in all entities.

### 6.1. Balance Factor of Losses

**Figure 6:**
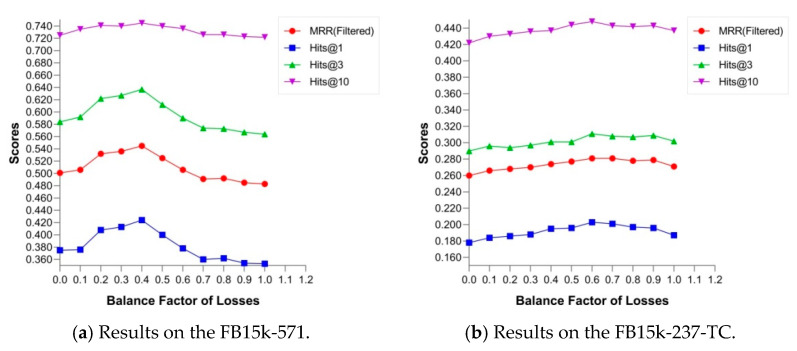
Effect of the strength of type constraints on the results of link prediction.

The impartment of type constraints was beneficial for capturing ontology-based associations between entities and correctly completing the missing entities in relations. To prove this point, we investigated the performance of link prediction on FB15K-571 and FB15K-237-TC with diverse values of the balance factor of losses α that indicated the strength of type constraints and reported the results in Figure 6. As shown in Figure 6, all metrics increased with the balance factor of losses when α increased from 0 to 0.4 on FB15K-571. However, most metrics started to decline after α reached 0.4. On FB15K-237-TC, the experimental results showed similar trends, and the identical inflection point $\alpha_{ip}=0.6$ was slightly different from the experiment on FB15K-571. Thus, from this investigation, we conceived that an appropriate balance factor of losses contributed to improve the performance of CHolE on link prediction, and excessive values of α caused overconstraint that led to the degradation of performance.

### 6.2. Base of Type Radius

**Figure 7:**
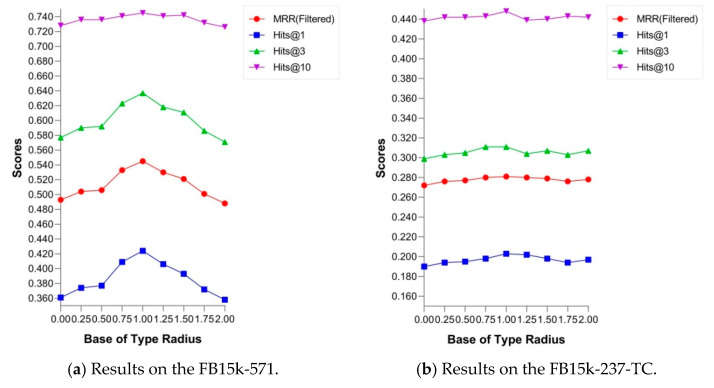
Effect of the scales of type constraints on the results of link prediction.

We discussed the effects of the strength of type constraints on the performance of link prediction in Section 6.1. Here, we implemented our model with the change in the base of type radius, a hyperparameter br in Equation (10), and reported the results of CHolE at different type constraint scales on two datasets (Figure 7). On the FB15K-571 dataset, most of the metrics increased with the base of type radius from 0 to 1 and decreased gradually when br>1. The experimental results on FB15K-237-TC reflected the similar tendencies of the performance change. The reason for this can be interpreted as follows: the suitable base of type radius (br=1) set type constraints to the appropriate scales that could make the constraints available, while possessing satisfactory distinguishing degrees for the entities owned by the same type. However, if the scale of constraints was set extremely small, then entities belonging to the same type could not be distinguished from each other. Excessively large base br can enlarge the scale of type constraints and cause partial or total invalidation of them.

## 7. Conclusions

In this paper, a novel complex embedding method called CHolE was proposed to extend the classic compositional representation HolE [17] to complex domain and model the entities and relations with their type constraints in the complex vector space. It encoded the type constraints and nonontological interactions as the modulus constraints and phase interactions of complex embeddings, respectively, and introduced the complex circular correlation to integrate them together and capture the multifaceted relatedness between entities in KGs. Thus, in comparison with previous complex embedding methods, CHolE made more efficient use of the moduli and phase angle of the complexes by taking them as two relatively independent degrees of freedom to encode the ontological information and nonontological information in KGs. Thus, type constraints can be well integrated into representational embeddings of entities and relations for improving the model’s performance on link prediction. The experimental results on benchmark datasets showed that the proposed method outperformed previous state-of-the-art methods, and the impartment of type constraints could improve the model’s performance on link prediction effectively. Moreover, this work also demonstrated the importance of the type information in KGs for some critical tasks, such as link prediction. Thus, efficient use of it will enable downstream applications in various fields to benefit more from KGs.

Nevertheless, the proposed method focused only on modeling type constraints rather than other ontology-level relations, such as hierarchical-aware relations. In the future, we plan to extend the type embeddings to complex vector space and construct hierarchical structures of types by improving the interaction mechanism of modulus and phase angles of complex embeddings.

## Figures and Tables

**Table 1:** Symbols and descriptions.

Symbols	Descriptions	Symbols	Descriptions
KG	knowledge graph	*r_TCR_*	TCR relation
E	entity set	S	triple set
C	type (concept) set	S*_I_*	general triple set
R	relation set	S*_TC_*	type constraint triple set
R*_I_*	instance-level relation set	S*_TCE_*	TCE triple set
R*_TC_*	type constraint relation set	S*_TCE_*	TCR triple set
*r_TCE_*	TCE (*instanceOf*) relation		

**Table 2:** Statistics of FB15K-571 and FB15K-237-TC.

Dataset	FB15K-571	FB15K-237-TC
#Entity *	14,951	14,541
#Type	571	542
#General (Instance-level) Relation	1345	237
#General Relation Triple	592,213	310,116
#TCE (*instanceOf* Relation) Triple	123,842	121,287
#TCR Triple	1345	237
#Train (General Relation Triple)	483,142	272,115
#Valid (General Relation Triple)	50,000	17,535
#Test (General Relation Triple)	59,071	20,466

* The #X represents the number of elements in the X set.

**Table 3:** Link prediction results on FB15K-571 and FB15K-237-TC *.

Dataset	FB15K-571					FB15K-237-TC				
Metrics	MRR		Hits@N			MRR		Hits@N		
Setting	Raw	Filter	N = 1	N = 3	N = 10	Raw	Filter	N = 1	N = 3	N = 10
TransE		0.417	0.150	0.314	0.476	0.144	0.233	0.147	0.263	0.398
TransH		0.495	0.284	0.535	0.641		0.136	0.041	0.160	0.331
RESCAL	0.189	0.354	0.235	0.409	0.587		0.255	0.185	0.278	0.397
DistMult		0.350			0.577	0.100	0.191	0.106	0.207	0.376
HolE	**0.232**	0.524	0.402	0.613	0.739	0.124	0.222	0.133	0.253	0.391
ComplEx	0.223	0.485	0.347	0.577	0.729	0.109	0.201	0.112	0.213	0.388
CHolE (RL only)	**0.232**	0.510	0.387	0.601	0.725	0.158	0.260	0.178	0.290	0.422
CHolE (TC+RL)	0.231	**0.543**	**0.424**	**0.637**	**0.746**	**0.185**	**0.281**	**0.203**	**0.311**	**0.448**

* Best score is in **bold**, and scores that are underlined represent better results than the original model HolE. For FB15K-571, the scores of DistMult [14] and HolE [17] are taken from the corresponding original papers, the results of TransE [11] and TransH [12] are taken from [39], and the result of RESACAL [15] comes from [17]. For FB15K-237-TC, the scores of TransE [11], DistMult [14], HolE [17], and ComplEx [16] are taken from [29], and the results of TransH [12] and RESCAL [15] come from [39,40], respectively.

## Data Availability

The dataset FB15K-571 investigated in this work are publicly available at https://github.com/thunlp/TKRL (accessed on 30 October 2015), and the publicly dataset FB15K-237 that is processed in this work to construct the dataset FB15K-237-TC can be found at https://www.microsoft.com/en-us/download/details.aspx?id=52312 (accessed on 19 February 2019).
